# Palpation of the Uterine Appendages

**Published:** 1901-04-13

**Authors:** 


### Hospital Clinics and Medical Progress.

### PALPATION OF THE UTERINE APPENDAGES.

Notwithstanding the perfection to which, by practice and education of the sense of touch, gynæcologists have carried the art of physical diagnosis in pelvic diseases, there is much reason for believing that to a not inconsiderable number of medical men, who are actively engaged in general practice, one of the points in diagnosis on which most stress is laid by gynæcologists, namely, palpation of the ovaries, remains practically a sealed book. Dr. George Gray Ward,[1] of New York, writing on this subject, says that he frequently meets such men. They can make a diagnosis as to the position of the uterus, the condition of the cervix, or the pelvic floor, but the condition of the ovary and tube is to them a "Will-o'-the-wisp," and he devotes a paper entirely to an explanation of the method to be adopted in order to overcome this difficulty. In conducting a bi-manual examination the patient should, he says, be placed in the dorsal position, preferably with the hips elevated, all tight clothing should be thoroughly loosened, and the thighs flexed upon the abdomen and rotated outwards. This position can be best attained by the use of the leg-holders devised by Professor Edebohls. In all cases the patient should empty her bladder before going on the table. The lower bowel also should be emptied. It is essential to have the fact firmly impressed on one's mind that more can be accomplished by gentleness of manipulation, and by skill, than by physical force, and that where it is necessary to use deep pressure it must be done slowly and gradually. The examiner should accustom himself to use either hand, as it is easier to reach the left appendage with the left hand, and the right appendage with the right hand. If necessary the thumb can be closed upon the index finger so that it passes under the pelvic arch. By this method the whole pelvic floor can be invaginated to a considerable extent, and a marked gain in the reach can be obtained. The elbow of the examining hand should rest against the examiner's hip, and all presure should be made by throwing the weight of the body upon the elbow, thus allowing the muscles of the forearm and arm to be at rest, which greatly facilitates the vaginal touch. The first step necessary to the palpation of the adnexa is to locate the fundus of the uterus. The index finger, or, if the vagina will admit, the index and middle fingers are gently and slowly passed into the vagina until the cervix is reached. The palmar surfaces should be uppermost, and the fingers should pass into the cul de sac beyond the cervix, so that that organ should rest upon their tips. Slight upward pressure in the direction of the inlet of the pelvis is then made, while the outer hand makes counter pressure on the abdominal wall midway between the umbilicus and the symphysis. The four fingers of the outer hand must be kept close together, and the palmar surfaces used rather than the tips. The fundus uteri will then be reached, as is indicated by the pressure on the uterus being at once communicated to the fingers in the vagina. The size and shape and mobility of the uterus can thus be estimated. All this of course is simple and everyday.

In cases of backward or forward displacement of the uterus, other manœuvres may be necessary in order to locate the fundus, but this once done one must remember that the tube and ovary, unless bound down by adhesions, are movable, being loosely attached to the horns of the uterus. In this fact lies the cause of the difficulty which some experience in reaching them. They must then be sought for, and it is best to let one hand, the external one, do the searching, while the other, the inner one, remains stationary for the time being to act as a counterpoint, each half of the pelvis being systematically gone over separately. For this purpose the vaginal finger is slipped to one side of the cervix, into the lateral fornix, and pressure is made in a direction upward and backward at a point midway between the cervix and the pelvic wall, while the four fingers of the external hand, curved in the shape of a scoop, and held close together, systematically rake the area from between the fundus and the side of the pelvis, moving in lines converging on the examining finger, or fingers, in the vagina, first commencing at the horn of the uterus and raking downwards until the fingers meet those of the vaginal hand, and repeating the process again and again, each time beginning a little nearer the side of the pelvis, until the whole area has been explored. The position of the vaginal fingers may then have to be varied, and the process repeated so as to cover new planes, but as soon as the ovary and tube are imprisoned between the scoop formed by the external hand and the fingers in the vagina, their character, size, sensibility and consistency can be made out as they slip between the opposing finger tips. The normal tube can thus be traced from the horn of the uterus. It feels like a cord about the diameter of a slate pencil and it is not painful to moderate pressure. The normal ovary feels as large as a walnut, and firm pressure gives the same sickening sensation and pain to the patient as pressure of the testicle. The opposite side of the pelvis can then be explored in a similar manner, using the right hand in the vagina for the right adnexa and *vice versâ*. In cases where the method above described does not succeed, resort should be had to recto-abdominal palpation, which is usually preferable when the uterus is retroverted or retroflexed, especially if the uterus is drawn down to the vaginal outlet by a tenaculum or volsellum. Recto-vagino-abdominal palpation may also be found useful in difficult cases, and where there is rigidity, much fat, or excessive sensitiveness, an anæsthetic may be necessary. While palpating the appendages a great deal can be learned concerning the condition of the broad ligaments; whether they are elastic and yielding to pressure, as they should be, or are dense, thickened, and resisting, as when adhesions are present, or there is plastic exudate.

[1] The Post Graduate, March 1901.

